# Antimycobacterial Mechanisms and Anti-Virulence Activities of Polyphenolic-Rich South African Medicinal Plants Against *Mycobacterium smegmatis*

**DOI:** 10.3390/microorganisms14010239

**Published:** 2026-01-20

**Authors:** Matsilane L. Mashilo, Mashilo M. Matotoka, Peter Masoko

**Affiliations:** Faculty of Science and Agriculture, Department of Biochemistry, Microbiology and Biotechnology, University of Limpopo, Private Bag X1106, Sovenga 0727, South Africa; lee.mashilo79@gmail.com (M.L.M.); mashilo.matotoka@ul.ac.za (M.M.M.)

**Keywords:** antimycobacterial, antibiofilm, antimotility, medicinal plants

## Abstract

The rise of multidrug-resistant tuberculosis (TB) necessitates alternative therapeutic sources. This study investigated the polyphenolic content and the antioxidant, antimycobacterial, and anti-virulence activities of selected medicinal plants traditionally used to treat TB and related symptoms. Total phenolics, tannins, and flavonoids were quantified using colorimetric assays. Antioxidant capacity was assessed via DPPH and ferric-reducing power assays. Antimycobacterial activity against *Mycobacterium smegmatis* was evaluated using broth microdilution, growth kinetics, cell constituent leakage, and respiratory chain dehydrogenase inhibition assays. Anti-virulence effects were examined using crystal violet biofilm and swarming motility assays. *Tarchonanthus camphoratus* showed the highest polyphenolic levels and, together with *Combretum hereroense*, strong antioxidant activity. Extracts of *Senecio macroglossus*, *Nerium oleander*, and *Tetradenia riparia* displayed potent antimycobacterial activity (MIC = 0.16 mg/mL), characterized by delayed exponential growth, membrane damage, and metabolic inhibition. *Tabernaemontana elegans* exhibited the weakest activity (MIC > 2.5 mg/mL). Most extracts also significantly impaired motility (12–100%) and early-stage biofilm formation. Polyphenolic-rich plant extracts demonstrated promising antimycobacterial and anti-virulence properties against *M. smegmatis*, highlighting their potential as leads for developing novel anti-TB agents.

## 1. Introduction

For thousands of years, tuberculosis (TB) has been a major infectious disease, continuing to cause high mortality, particularly among vulnerable populations. In 2023, an estimated 10.8 million people contracted TB, and 1.25 million died from the disease, according to the World Health Organization [1]. The TB incidence rate rose by 0.2% in 2023 compared to 2022. Globally, the TB incidence rate dropped by 8.3% between 2015 and 2023, which is far below the WHO End TB Strategy target of a 50% reduction by 2025. Meanwhile, the global TB death toll decreased by 23% over the same period, reaching nearly one-third of the 75% reduction goal set for 2025 [1].

TB is a chronic bacterial infection caused by members of the *Mycobacterium tuberculosis* complex, which includes *M. africanum*, *M. microti*, *M. tuberculosis*, and *M. bovis* [2]. The disease primarily affects the lungs but can involve other organs. Transmission occurs via airborne particles when an infected person coughs, sneezes, or speaks [3]. For the past five decades, drug-susceptible TB has been treated with a standardized six-month regimen developed from trials conducted between 1946 and 1986. This regimen includes a two-month intensive phase with rifampicin, isoniazid, and pyrazinamide, followed by four months of rifampicin and isoniazid. The addition of pyrazinamide reduced the treatment duration from nine to six months [4].

One emerging challenge in TB treatment is the persistence of tubercle bacilli, which reduces the efficacy of first-line drugs. *M. tuberculosis* employs multiple mechanisms to survive, including intracellular residence within macrophages and biofilm formation. Biofilm formation is a common bacterial survival strategy, and several mycobacterial species, including *M. marinum*, *M. fortuitum*, and *M. smegmatis*, can form biofilms through quorum sensing [5,6,7].

Given the rise of drug-resistant TB strains and reduced treatment efficacy, there is an urgent need to identify compounds with novel mechanisms of action against *M. tuberculosis*. Medicinal plants have historically provided a rich source of bioactive compounds, and many modern drugs are derived from natural sources. Plant-derived compounds offer diverse functional groups, multiple mechanisms of action, minimal drug resistance, and limited side effects [8,9]. Hence, developing effective plant-based anti-TB agents could help overcome resistance and reduce TB-related morbidity and mortality [10].

The herbal plants used in traditional medicine have contributed to many modern drugs and are generally associated with minimal side effects. It is estimated that over 70% of medicinal drugs originate from natural sources, and more than 80% of the global population relies on plant-based health products [11]. The selection of plants in this study (*Agapanthus praecox*, *Combretum hereroense*, *Leonotis ocymifolia*, *Nerium oleander*, *Polygala myrtifolia*, *Olea europaea* subsp. *africana*, *Senecio macroglossus*, *Tabernaemontana elegans*, *Tarchonanthus camphoratus*, and *Tetradenia riparia*) was guided by their ethnopharmacological use in treating TB and related symptoms [12,13,14].

Despite documentation of their antimycobacterial, antioxidant, and polyphenolic properties, there is a notable gap in understanding the mechanisms of action and anti-virulence activities of these plants. In this study, *Mycobacterium smegmatis* was used as a model organism. While it differs from *M. tuberculosis* in pathogenicity, virulence, and metabolism, it shares a highly similar cell wall structure, which is the primary target of the cell-rupture-based mechanisms investigated in this work. The use of *M. smegmatis* facilitates the safe and efficient exploration of mycobacterial physiological mechanisms under controlled laboratory conditions, serving as a suitable surrogate for pathogenic species due to its conserved cell wall architecture. Consequently, this study aimed to evaluate the polyphenolic profiles, antioxidant capacities, and antimycobacterial activities of selected plant extracts. Furthermore, we investigated their interference with biofilm-associated virulence properties and sought to elucidate the underlying mechanisms of their antimycobacterial action, specifically focusing on membrane integrity and metabolic suppression.

## 2. Materials and Methods

### 2.1. Chemicals and Reagents

The following analytical-grade chemicals and reagents were used: hexane, dichloromethane, acetone, methanol, Folin–Ciocalteu reagent, sodium carbonate, aluminum chloride, L-ascorbic acid, sodium nitrite, hydrogen peroxide, sodium acetate, quercetin, gallic acid, 2,2-diphenyl-1-picrylhydrazyl (DPPH), potassium ferricyanide, trichloroacetic acid, ferric chloride, middlebrook 7H9 broth, and *p*-iodonitrotetrazolium chloride (all from Merck, Darmstadt, Germany); glycerol and oleic albumin dextrose catalase (OADC) supplement (Fluka, Buchs, Switzerland); Bradford reagent and glucose (Sigma-Aldrich, St. Louis, MO, USA); and bacteriological agar (LabM, Heywood, UK). Whatman No. 1 filter paper was purchased from Sigma-Aldrich (USA).

### 2.2. Plant Collection

The selection of plants was based on their ethnopharmacological use in the treatment of tuberculosis and related symptoms. Leaves of *Agapanthus praecox* (UNIN 1220272), *Combretum hereroense* (UNIN 1220269), *Leonotis ocymifolia* (UNIN 1220259), *Nerium oleander* (UNIN 1220282), *Polygala myrtifolia* (UNIN 1220275), *Olea europaea subsp. africana* (UNIN 1220261), *Senecio macroglossus* (UNIN 1220273), *Tabernaemontana elegans* (UNIN 1220268), *Tarchonanthus camphoratus* (UNIN 1220260), and *Tetradenia riparia* (UNIN 1220257) were collected from the University of Limpopo Botanical Garden and the Lowveld National Botanical Garden (Mpumalanga, Mbombela, South Africa). Plant identification was confirmed by Dr. Egan Bronwyn, and voucher specimens were deposited at the Larry Leach Herbarium, University of Limpopo (Polokwane, South Africa). Leaves were air-dried in the dark at room temperature for two weeks, finely ground, and stored in airtight containers under ambient laboratory conditions.

### 2.3. Extraction of Plant Material

One gram of the finely ground plant material was placed into each 50 mL centrifuge tube, followed by 10 mL of solvent (hexane, dichloromethane, acetone, methanol, or water) (all SupraSolv^®^, Darmstadt, Germany). Tubes were shaken at 200 rpm for 30 min using a shaking incubator (New Brunswick Scientific Co., Inc., Edison, NJ, USA). Extracts were filtered through filter paper into pre-weighed glass vials, and solvents were evaporated using a fan. Extract yield was calculated by subtracting the weight of the empty vial from that of the dried extract. The dried extracts were dissolved in acetone to obtain a stock concentration of 10 mg/mL for further assays.

### 2.4. Quantification of Polyphenolics

#### 2.4.1. Total Phenolic Content (TPC)

The TPC was determined using the Folin–Ciocalteu method described by Tambe and Bhambar [15] with minor modifications. A 100 µL aliquot of each extract (10 mg/mL) was diluted with 900 µL distilled water and mixed with 250 µL Folin–Ciocalteu reagent (Sigma-Aldrich^®^, St. Louis, MO, USA). After 5 min, 1.25 mL of 7% sodium carbonate solution (Sigma-Aldrich^®^, St. Louis, MO, USA) was added, and the mixture was incubated in the dark at room temperature for 30 min. Absorbance was read at 725 nm using a UV–VIS spectrophotometer (Genesys 10S UV-VIS, Menlo Park, CA, USA). Gallic acid was used to construct a standard curve (0.08–1.25 mg/mL), and results are expressed as mg gallic acid equivalents per gram of extract (mg GAE/g). The experiment was conducted in triplicate and independently repeated three times. The total phenolic content (TPC) was calculated using the gallic acid calibration curve (y = 0.6918x + 0.0039; R^2^ = 0.9976).

#### 2.4.2. Total Tannin Content (TTC)

Tannin content was quantified using the Folin–Ciocalteu method by Tambe and Bhambar [15]. Briefly, 50 µL of each extract (10 mg/mL) was mixed with 3.8 mL distilled water and 0.25 mL Folin–Ciocalteu reagent (Sigma-Aldrich^®^, St. Louis, MO, USA), followed by 0.5 mL of 35% sodium carbonate solution (Sigma-Aldrich^®^, St. Louis, MO, USA). The mixture was diluted to 10 mL with distilled water, vortexed, and incubated in the dark for 30 min. Absorbance was measured at 725 nm using a UV–VIS spectrophotometer (Genesys 10S UV-VIS, Menlo Park, CA, USA). Gallic acid (Sigma-Aldrich^®^, St. Louis, MO, USA) was used as a standard (0.0625–1 mg/mL), and results are expressed as mg GAE/g extract. The experiment was conducted in triplicate and independently repeated three times. The total tannin content (TTC) was calculated using the gallic acid calibration curve (y = 0.7918x + 0.049, R^2^ = 0.9785).

#### 2.4.3. Total Flavonoid Content (TFC)

Flavonoid content was determined using the aluminum chloride colorimetric method by Tambe and Bhambar [15]. Each extract (100 µL of 10 mg/mL) was mixed with 4.9 mL of distilled water and 300 µL of 5% sodium nitrite (Supelco^®^, Bellefonte, PA, USA). After 5 min, 300 µL of 10% aluminum chloride (Sigma-Aldrich^®^, St. Louis, MO, USA) was added, followed by 2 mL of 1 M sodium hydroxide (Supelco^®^, Bellefonte, PA, USA) after another 5 min. The final volume was adjusted to 10 mL with distilled water. Absorbance was measured at 510 nm using a UV–VIS spectrophotometer (Genesys 10S UV-VIS, Menlo Park, CA, USA). Quercetin (Sigma-Aldrich^®^, St. Louis, MO, USA) was used as a standard (31.25–500 µg/mL). Total flavonoid content is expressed as mg QE/g extract. The experiment was conducted in triplicate and repeated three times. The flavonoid content was calculated using the equation from the quercetin standard curve (y = 0.1129x + 0.0043).

#### 2.4.4. Total Flavonol Content (TFlC)

The total flavonol content was determined following Iqbal et al. [16]. Extracts (1 mg/mL) were mixed with 0.5 mL of 2% aluminum chloride (Sigma-Aldrich^®^, St. Louis, MO, USA) and 1.5 mL of 5% sodium acetate. Mixtures were vortexed, centrifuged, and absorbance was measured at 440 nm using a UV–VIS spectrophotometer (Genesys 10S UV-VIS, Menlo Park, CA, USA). Quercetin (Sigma-Aldrich^®^, St. Louis, MO, USA) served as the standard (16–250 µg/mL), and results are expressed as mg QE/g extract. The experiment was conducted in triplicate and repeated three times. The flavonol content was calculated using the equation from the quercetin standard curve (y = 0.209x + 0.0033).

### 2.5. Quantitative Antioxidant Activity

#### 2.5.1. DPPH Free Radical Scavenging Assay

The DPPH radical scavenging activity of the extracts was evaluated according to Chigayo et al. [17]. Extracts (15.63–250 µg/mL) were prepared in methanol. To each 1 mL extract, 2 mL of 0.2 mM DPPH (Sigma-Aldrich^®^, St. Louis, MO, USA) methanolic solution was added, vortexed, and incubated in the dark at room temperature for 30 min. A control was prepared by mixing 2 mL of 0.2 mmol/L DPPH with 1 mL of methanol (SupraSolv^®^, Darmstadt, Germany). Absorbance was measured at 517 nm using a UV–VIS spectrophotometer (Genesys 10S UV-VIS, Menlo Park, CA, USA). L-ascorbic acid (Sigma-Aldrich^®^, St. Louis, MO, USA) served as a positive control.

The percentage antioxidant potential of the solution was calculated using the following formula:

$$\%Inhibition=\frac{Ac-As}{Ac}\times 100$$

where Ac is the absorbance of the control solution, and As is the absorbance of the plant extract. The experiment was performed in triplicate.

#### 2.5.2. Ferric-Reducing Power Assay

The ferric-reducing antioxidant power (FRAP) of the plant extracts was determined following the method of Vijayalakshmi and Ruckmani [18]. A 1250 µg/mL stock solution was serially diluted to obtain five concentrations (625–39 µg/mL). For each concentration, 2.5 mL of the extract was mixed with 2.5 mL of 0.2 M sodium phosphate buffer (pH 6.6) (Sigma-Aldrich^®^, St. Louis, MO, USA) and 2.5 mL of 1% potassium ferricyanide (Sigma-Aldrich^®^, St. Louis, MO, USA) solution, followed by vortexing. The mixtures were incubated at 50 °C for 20 min, after which 2 mL of 10% trichloroacetic acid (Sigma-Aldrich^®^, St. Louis, MO, USA) was added. Subsequently, 5 mL of the resulting supernatant was transferred to a clean test tube and combined with 5 mL of distilled water and 1 mL of 0.1% ferric chloride solution, vortexing after each addition. Absorbance was measured at 700 nm using a UV–VIS spectrophotometer (Genesys 10S UV-VIS, Menlo Park, CA, USA). A blank was prepared using acetone instead of the extract. L-ascorbic acid (39–625 µg/mL) served as the positive control. All experiments were conducted in triplicate.

### 2.6. Antimycobacterial Activity

#### 2.6.1. Microorganism Used in This Study

*Mycobacterium smegmatis* was kindly provided by Prof. Green (Department of Biotechnology and Food Technology, University of Johannesburg, Gauteng, South Africa). The strain was maintained on Middlebrook 7H9 agar supplemented with glycerol and Middlebrook oleic albumin dextrose catalase (OADC) growth supplement. For experimental use, the bacterium was cultured in Middlebrook 7H9 broth containing glycerol and OADC and incubated at 37 °C for 24 h.

#### 2.6.2. Microdilution Assay

The minimum inhibitory concentration (MIC) of each extract (2.5–0.019 mg/mL) was determined using the serial microplate broth dilution method described by Eloff [19]. Plant extracts were dissolved in acetone to a final concentration of 10 mg/mL. In a sterile 96-well microtiter plate, 100 µL of sterile distilled water was added to all wells. Then, 100 µL of each extract was added to the first well and serially diluted two-fold (1:1) across the plate. An equal volume (100 µL) of *M. smegmatis* inoculum (~1 × 10^6^ cfu/mL) was added to each well. Rifampicin (Sigma-Aldrich^®^, St. Louis, MO, USA) served as the positive control, while acetone served as the negative control. Plates were sealed and incubated at 37 °C for 24 h.

After incubation, 40 µL of *p*-iodonitrotetrazolium violet (INT; 0.2 mg/mL in water) (Sigma-Aldrich^®^, St. Louis, MO, USA) was added to each well, followed by a further 30 min incubation at 37 °C under humid conditions. The MIC was recorded as the lowest concentration showing no color change (indicating absence of bacterial growth). Minimum bactericidal concentrations (MBCs) were determined by extending incubation for an additional 24 h at 37 °C. All assays were performed in triplicate. Total activity was calculated by dividing the extract yield (mg) by its MIC value (mg/mL).

#### 2.6.3. Combinational Effects of Plant Extracts

The combinational effects of the plant extracts were assessed according to the method of van Vuuren and Viljoen [20] to determine the fractional inhibitory concentration (FIC). The broth microdilution assay was performed as described above.

The FIC for each extract was calculated using Equation (1):

(1) $${\mathrm{FIC}}_{A}=\frac{\mathrm{MIC}\;\mathrm{of}\;A\;\mathrm{in}\;\mathrm{combination}}{\mathrm{MIC}\;\mathrm{of}\;A\;\mathrm{alone}},{\mathrm{FIC}}_{B}=\frac{\mathrm{MIC}\;\mathrm{of}\;B\;\mathrm{in}\;\mathrm{combination}}{\mathrm{MIC}\;\mathrm{of}\;B\;\mathrm{alone}}$$

The sum of FICs (ΣFIC) was obtained using Equation (2):

(2) $$\Sigma \mathrm{FIC}={\mathrm{FIC}}_{A}+{\mathrm{FIC}}_{B}$$

The interaction was interpreted as synergistic (ΣFIC ≤ 0.5), additive (ΣFIC > 0.5–1.0), indifferent (ΣFIC > 1.0–4.0), or antagonistic (ΣFIC > 4.0).

#### 2.6.4. Evaluation of Growth Kinetics During Treatment

The growth kinetics of *M. smegmatis* were evaluated over 24 h in the presence of plant extracts and rifampicin (positive control) (Sigma-Aldrich^®^, St. Louis, MO, USA), as described by Jiang et al. [21]. The bacterium was inoculated into Middlebrook 7H9 broth containing 2% glucose (Sigma-Aldrich^®^, St. Louis, MO, USA), glycerol (Sigma-Aldrich^®^, St. Louis, MO, USA), and 0.25% Tween 80, and incubated at 37 °C for 24 h. Overnight cultures were transferred into 20 mL of Middlebrook 7H9 broth supplemented with the test extracts or rifampicin, and the initial optical density (OD_600_) was adjusted to 0.02 (~1 × 10^6^ cfu/mL). Cultures were incubated at 37 °C with continuous shaking, and bacterial growth was monitored by measuring OD_600_ at 3, 6, 9, 18, and 24 h using a UV–VIS spectrophotometer (Genesys 10S UV-VIS, Menlo Park, CA, USA). An untreated culture served as the negative control, and media-only served as a blank.

### 2.7. Antimycobacterial Mechanism of the Plant Extracts

#### 2.7.1. Measurement of Intracellular Protein and DNA Leakage

The potential of the plant extracts to induce intracellular protein and DNA leakage in *M. smegmatis* was assessed following Machingauta and Mukanganyama [22], with slight modifications. Overnight bacterial cultures were harvested by centrifugation and resuspended in phosphate-buffered saline (PBS; pH 7.2) (Sigma-Aldrich^®^, St. Louis, MO, USA) to achieve an OD_600_ = 0.5. Two-fold serial dilutions of the extracts were prepared to obtain concentrations corresponding to the MIC to ¼ MIC, which were then added to the bacterial suspensions. Rifampicin (Sigma-Aldrich^®^, St. Louis, MO, USA) served as a positive control, while PBS and untreated bacterial cultures served as negative controls. Samples were incubated at 37 °C with shaking (150 rpm) for 2 h, then centrifuged at 14,000 rpm for 3 min.

For DNA leakage, 50 μL of the supernatant was transferred into a 96-well microtiter plate, and absorbance was read at 260 nm using a Promega microplate reader. For protein leakage, 50 μL of Bradford reagent was added to the supernatant, incubated for 5 min at room temperature, and measured at 595 nm using a UV–VIS spectrophotometer (Genesys 10S UV-VIS, Menlo Park, CA, USA). The percentage of leakage was calculated using the following formula:

$$\mathrm{Leakage}\;(\%)=\frac{OD_{x}-OD_{c}}{OD_{c}}\times 100$$

where ODₓ and ODc represents the absorbance of the treated and control samples, respectively.

#### 2.7.2. Measurement of INT-Dehydrogenase Relative Activity

The INT-dehydrogenase relative activity (DRA) assay was performed as described by Ding et al. [23] and Ersoy et al. [24], with modifications. Briefly, bacterial pellets (0.2 mL) suspended in PBS (pH 7.2) were treated with 0.2 mL of test samples at MIC, ½ MIC, and ¼ MIC concentrations, and incubated at 37 °C for 2 h. Rifampicin served as the positive control, while PBS and untreated cultures served as negative controls.

After incubation, 100 μL of INT-glucose solution (0.4% INT and 0.1 mol/L glucose) (Sigma-Aldrich^®^, St. Louis, MO, USA) was added and incubated for 1 h at 37 °C to allow color development. The mixture was centrifuged at 14,000 rpm for 5 min, and 50 μL of the supernatant was transferred to a 96-well plate, followed by the addition of 50 μL of absolute ethanol (SupraSolv^®^, Darmstadt, Germany). The mixture was shaken for 3 min to dissolve the formazan, and absorbance was measured at 485 nm using a UV–VIS spectrophotometer (Genesys 10S UV-VIS, Menlo Park, CA, USA). The DRA was calculated as

$$\mathrm{DRA}\;(\%)=\frac{OD_{x}}{OD_{c}}\times 100$$

where *ODc* and *ODₓ* denote the absorbance of control and treated samples, respectively.

### 2.8. Antibiofilm Activity Assays

#### 2.8.1. Prevention of Initial Cell Attachment

The inhibition of initial cell attachment was evaluated following the method of Famuyide et al. [25]. A 100 μL aliquot of *M. smegmatis* culture (OD_600_ = 0.02; ~1 × 10^6^ cfu/mL) was added to individual wells of a flat-bottomed 96-well microtiter plate and incubated at 37 °C for 4 h without agitation. After incubation, 100 μL of the plant extract (in acetone) at different concentrations (MIC, ½ MIC, ¼ MIC, and ⅛ MIC) was added to each well and further incubated for 24 h at 37 °C. Rifampicin (Sigma-Aldrich^®^, St. Louis, MO, USA) served as the positive control, while acetone (SupraSolv^®^, Darmstadt, Germany) and 7H9-glucose broth (Sigma-Aldrich^®^, St. Louis, MO, USA) were used as negative controls. Biofilm formation was quantified using the crystal violet staining assay. The experiment was conducted in triplicate.

#### 2.8.2. Prevention of Biofilm Formation

To assess biofilm formation inhibition, 100 μL of bacterial suspension (OD_600_ = 0.02; ~1 × 10^6^ cfu/mL) was co-incubated with 100 μL of plant extracts (MIC–⅛ MIC) in 96-well plates at 37 °C for 24 h. Untreated culture and media-only were used as negative controls, while rifampicin (Sigma-Aldrich^®^, St. Louis, MO, USA) was used as a positive control. The experiment was conducted in triplicate. Biofilm biomass was then quantified using crystal violet staining.

#### 2.8.3. Eradication of Pre-Formed Biofilms

To evaluate the eradication of mature biofilms, *M. smegmatis* cultures (OD_600_ = 0.02; ~1 × 10^6^ cfu/mL) were first incubated for 24 h at 37 °C to allow biofilm formation. The planktonic cells were discarded, and 100 μL of plant extracts (MIC–⅛ MIC) was added, followed by a further 24 h incubation at 37 °C. The remaining biofilm biomass was quantified using the crystal violet assay. The experiment was conducted in triplicate. Untreated culture and media-only were used as negative controls, while rifampicin (Sigma-Aldrich^®^, St. Louis, MO, USA) was used as a positive control.

#### 2.8.4. Crystal Violet Staining Assay

Biofilm biomass was quantified using a modified crystal violet (CV) staining method. After incubation, wells were washed three times with sterile distilled water, air-dried, and then oven-dried at 60 °C for 45 min. Each well was stained with 100 μL of 1% CV (Sigma-Aldrich^®^, St. Louis, MO, USA) for 15 min at room temperature, washed three times, and air-dried. Subsequently, 100 μL of methanol was added to solubilize the bound dye, and absorbance was read at 595 nm using a UV–VIS spectrophotometer (Genesys 10S UV-VIS, Menlo Park, CA, USA). The percentage of biofilm inhibition was calculated as follows [26]:

$$\mathrm{Biofilm}\;\mathrm{inhibition}\;(\%)=\frac{OD_{\mathrm{control}}-OD_{\mathrm{treated}}}{OD_{\mathrm{control}}}\times 100$$

where OD_control_ is the migration diameter of untreated bacteria, and OD_treatment_ is that of extract-treated cultures.

### 2.9. Antimotility Activity

The antimotility (anti-swarming) effect of the extracts was assessed following the method of Caigoy et al. [27]. Swarming agar was prepared using Middlebrook 7H9 broth supplemented with 0.5% bacteriological agar (Sigma-Aldrich^®^, St. Louis, MO, USA). The plates containing MIC and ½ MIC concentrations of plant extracts, rifampicin (Sigma-Aldrich^®^, St. Louis, MO, USA). as the positive control, and dimethyl sulfoxide (SupraSolv^®^, Darmstadt, Germany) used as a negative control were spot-inoculated at the center with 5 μL of an overnight culture. Plates were incubated at 37 °C, and colony migration was measured. Antimotility activity was calculated using

$$\mathrm{Antimotility}\;(\%)=\frac{D_{\mathrm{control}}-D_{\mathrm{treatment}}}{D_{\mathrm{control}}}\times 100$$

where D_control_ is the migration diameter of untreated bacteria, and D_treatment_ is that of extract-treated cultures.

### 2.10. Statistical Analysis

All experiments were conducted in triplicate, and results are expressed as mean ± standard deviation (SD). Statistical significance was determined using one-way ANOVA followed by Dunnett’s post hoc test (GraphPad Prism version 9.0). Differences were considered statistically significant at *p* < 0.05.

## 3. Results

### 3.1. Extract Mass Obtained from Plant Material

**Figure 1:**
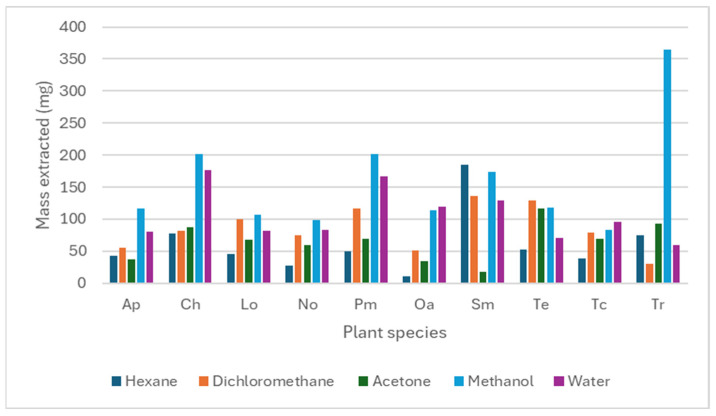
Mass of the plant extracts extracted using different solvents. Ap—*Agapanthus praecox*, Ch—*Combretum hereroense*, Lo—*Leonotis ocymifolia*, No—*Nerium oleander*, Pm—*Polygala myrtifolia*, Oa—*Olea europaea* subsp. *africana*, Sm—*Senecio macroglossus*, Te—*Tabernaemontana elegans*, Tc—*Tarchonanthus camphoratus*, and Tr—*Tetradenia raparia*.

Figure 1 shows the mass (mg) of extract obtained from 1 g of powdered plant material using five different solvents. Methanol and water were the most efficient extractants, with the methanolic extract of *T. riparia* yielding 364 mg/g of plant material. Conversely, hexane yielded the lowest extraction efficiency, with *O. africana* producing only 10 mg/g.

### 3.2. Total Polyphenolic Content of Acetone Plant Extracts

*T. camphoratus* exhibited the highest total phenolic, tannin, and flavonoid contents, with values of 28.94 ± 0.53 mg GAE/g, 2.019 ± 0.009 mg GAE/g, and 30.61 ± 0.09 mg QE/g, respectively. By contrast, *T. elegans* showed the highest flavonol content (43.19 ± 0.24 mg QE/g) (Table 1).

### 3.3. Antioxidant Activity of the Different Plant Extracts

Antioxidant evaluation showed that *T. camphoratus* and *C. hereroense* exhibited the strongest antioxidant activity in both assays, whereas *T. elegans* showed the weakest activity. In the DPPH assay, the extract of *T. camphoratus* displayed the highest radical-scavenging activity, with an EC_50_ value of 38.73 µg/mL, while *T. elegans* showed the lowest activity (EC_50_ = 1456 µg/mL). In the ferric-reducing power assay, *C. hereroense* demonstrated the greatest reducing capacity (EC_50_ = 63.15 µg/mL), followed by *T. camphoratus*. In contrast, *T. elegans* exhibited the weakest reducing capacity, with an EC_50_ value of 2058 µg/mL (Table 2).

### 3.4. Antimycobacterial Activity Screening

The acetone and aqueous extracts of *S. macroglossus*, the aqueous extract of *N. oleander*, and the dichloromethane extract of *T. raparia* exhibited the strongest antimycobacterial activity, with MIC values of 0.16 mg/mL. In contrast, the aqueous extract of *T. elegans* showed the lowest activity, with MIC values exceeding 2.5 mg/mL. All extracts displayed MBC values > 2.5 mg/mL, indicating the absence of bactericidal activity under the tested conditions. Furthermore, the aqueous extracts of *S. macroglossus* and *N. oleander* demonstrated the highest total activity values of 806.25 and 518.75 mL/g, respectively (Table 3).

### 3.5. Combinational Effects

The combinational effects among the plant extracts were predominantly indifferent, with only two combinations: *T. elegans* with *A. praecox* and *T. elegans* with *N. oleander* exhibiting additive interactions. Similarly, combinations of plant extracts with rifampicin mainly showed indifferent or antagonistic effects, with an additive effect observed only for the combination of *T. camphoratus* and rifampicin (Table 4).

### 3.6. Growth Kinetics

**Figure 2:**
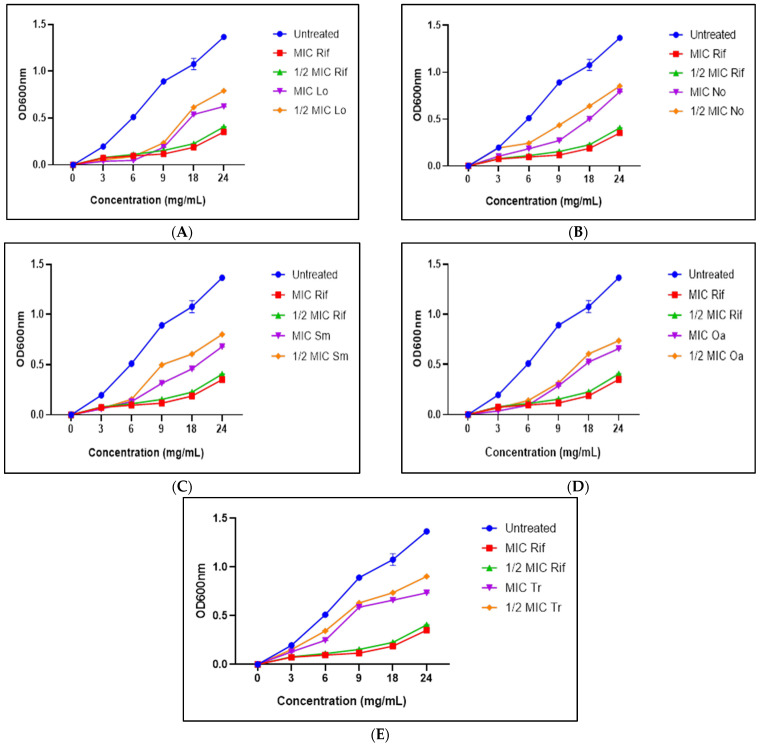
The effect of acetone extracts of (**A**) *L. ocymifolia*; (**B**) *N. oleander*; (**C**) *S. macroglossus*; (**D**) *O. europaea* supsp *africana*; (**E**) *T. raparia* on the growth of *Mycobacterium smegmatis* over a period of 24 h. Rif—rifampicin, Lo—*Leonotis ocymifolia*, No—*Nerium oleander*, Oa—*Olea europaea* supsp. *africana*, Sm—*Senecio macroglossus*, Tr—*Tetradenia raparia*, MIC—minimum inhibitory concentration.

All extracts significantly reduced bacterial growth compared with the control; however, none completely inhibited growth, as no treatment resulted in a sustained plateau or decline in optical density (Figure 2). Notably, *L. ocymifolia* at its MIC delayed the onset of the exponential growth phase until approximately 9 h of incubation, an effect comparable to that observed with rifampicin.

### 3.7. Mechanisms of Antimicrobial Action of Selected Extracts

**Figure 3:**
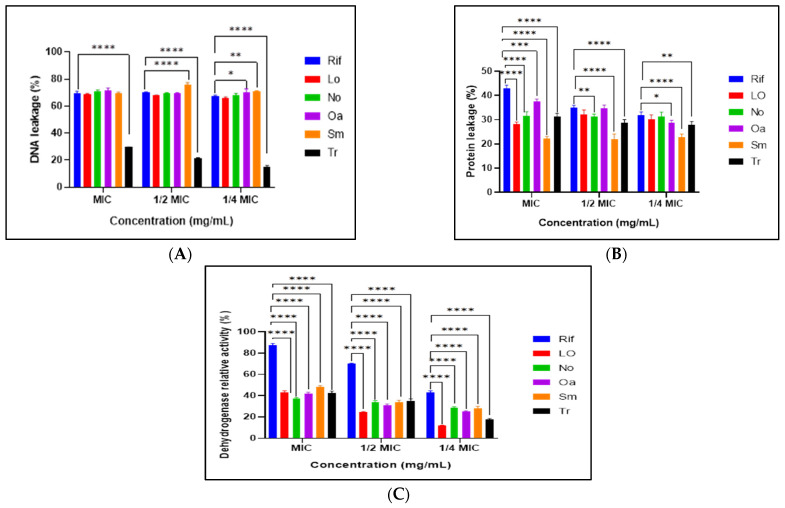
DNA leakage (**A**), protein leakage (**B**), and dehydrogenase relative activity (%) (**C**) of *M. smegmatis* cells following treatment with various concentrations of the plant extracts. Statistically significant differences were determined using two-way ANOVA coupled with Dunnett’s multiple-comparison test (*): *p* < 0.01; (**): *p* < 0.003; (***): *p* = 0.001; (****): *p* < 0.0001, Rif—rifampicin, Lo—*Leonotis ocymifolia*, No—Nerium *oleander*, Oa—*Olea europaea* supsp. *africana*, Sm—*Senecio macroglossus*, Tr—*Tetradenia raparia,* MIC—minimum inhibitory concentration.

The findings indicated a rise in the leakage of DNA and proteins, indicating potential damage to the cell membrane. Notably, DNA leakage exceeded 60% in all the plant extracts except for one plant, *T. raparia*, which exhibited a leakage range of 15% to 29%. In contrast, the protein leakage percentages remained below 40% across all plant extracts. All plant extracts demonstrated significant dehydrogenase inhibitory activity, indicating a marked reduction in the metabolic activity of the treated bacterial cells. Notably, *L. ocymifolia* exhibited a relative activity of 11% at ¼ MIC (Figure 3).

### 3.8. Antibiofilm Activity Assay

**Figure 4:**
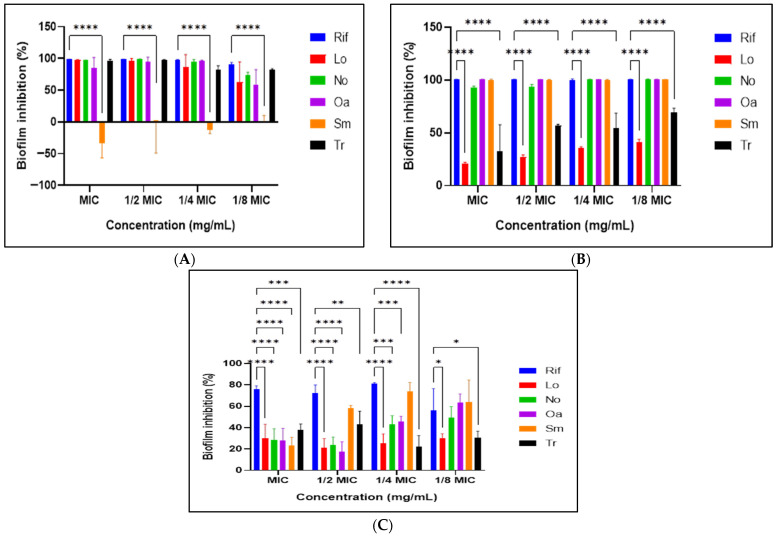
Inhibition of initial cell attachment (**A**), prevention of biofilm (**B**), and eradication of pre-formed biofilm (**C**) by the acetone extracts against *M. smegmatis*. Two-way ANOVA coupled with Dunnett’s multiple-comparison test was used to assess the statistical significance of differences (*): *p* < 0.01; (**): *p* < 0.003; (***): *p* = 0.001; (****): *p* < 0.0001, Rif—rifampicin, Lo—*Leonotis ocymifolia*, No—*Nerium oleander*, Oa—*Olea europaea* supsp. *africana*, Sm—*Senecio macroglossus*, Tr—*Tetradenia raparia,* MIC—minimum inhibitory concentration.

Significantly, the majority of the plant extracts exhibited considerable antibiofilm activity during the initial phase of cell attachment (62% to 98% inhibition rate) and the prevention stage of biofilm formation (93% to 100% inhibition rate) for the best extracts (Figure 4). Notably, *S. macroglossus* had higher eradication potential even at sub-MICs.

### 3.9. Antimotility Activity of the Selected Plant Extracts

The antimotility assay revealed variable inhibitory effects among the tested extracts against *M. smegmatis* (Table 5). Rifampicin completely inhibited motility at both MIC and ½ MIC (100%). Among the plant extracts, *N. oleander* showed complete inhibition at MIC (100%), while *T. raparia* exhibited near-complete inhibition at ½ MIC (99.29 ± 1.01%). Moderate antimotility activity was observed for *L. ocymifolia* and *O. europaea* subsp. *africana*, whereas *S. macroglossus* displayed higher inhibition at ½ MIC than at MIC.

## 4. Discussion

The present study evaluated the extraction efficiency, phytochemical composition, antioxidant capacity, and antimycobacterial activity of selected medicinal plants. Furthermore, it investigated biofilm-associated anti-virulence effects, providing insight into the biological relevance of these species and supporting their traditional therapeutic use. Extraction yields varied substantially across solvents, reflecting differences in solvent polarity and the solubility of specific plant metabolites. Methanol and water produced the highest extraction yields, with *T. riparia* (methanol) yielding up to 364 mg/g of plant material. This observation is consistent with established extraction principles, whereby polar solvents efficiently recover phenolics, flavonoids, anthocyanins, saponins, lipids, fatty acids, and vitamins, while aqueous solvents preferentially extract highly polar constituents such as polysaccharides and phenolic acids [28,29,30]. In contrast, hexane consistently produced the lowest yields, consistent with its selective extraction of non-polar compounds such as terpenes and fatty acids [31].

Quantitative phytochemical profiling revealed notable inter-species variation. *T. camphoratus* exhibited the highest total phenolic, tannin, and flavonoid contents, indicating a rich polyphenolic composition. Conversely, *T. elegans* showed comparatively lower concentrations of most phytochemical classes, consistent with previously reported low flavonoid concentrations (1.7 ± 0.1 mg QE/g extract) by Mnyambo [32]. Phenolic compounds are widely recognized for their antioxidant, antimicrobial, and anti-inflammatory properties [33], while tannins and flavonoids contribute to microbial growth modulation and membrane interactions [34,35,36].

Antioxidant assessment using the DPPH and FRAP assays demonstrated strong radical-scavenging and reducing capacities for *T. camphoratus* and *C. hereroense*, with EC_50_ values comparable to ascorbic acid. Discrepancies with earlier reports based on essential oil analysis [37] likely reflect methodological differences; methanolic extracts are enriched in polar antioxidants that are largely absent from volatile fractions. A previous study demonstrated that combining *Capparis sepiaria* roots with *T. elegans* bark resulted in modest ferric-reducing activity [38], supporting the relatively lower antioxidant potential of T. elegans observed in this study. The strong correlation observed between phenolic content and antioxidant activity supports the role of these compounds as primary contributors to redox modulation through hydrogen or electron donation [39].

Although oxidative stress is a recognized feature of mycobacterial infections and antimicrobial exposure [40], the antioxidant findings in this study are interpreted as supportive rather than directly therapeutic. Extracts with strong radical-scavenging activity, such as *T. camphoratus* and *C. hereroense*, may provide auxiliary benefits by mitigating oxidative stress and enhancing cellular resilience when considered alongside antimicrobial and antibiofilm effects.

Antimycobacterial activity was assessed using *M. smegmatis* as a non-pathogenic screening model. This organism is widely employed due to its rapid growth, genetic tractability, and conserved lipid-rich cell envelope architecture, a key antimicrobial target [41,42]. While *M. smegmatis* differs from *M. tuberculosis* in virulence regulation, it remains suitable for evaluating growth inhibition, membrane disruption, and metabolic interference. In this study, MIC values below 100 µg/mL were considered significant, 100–625 µg/mL moderate, and above 625 µg/mL low activity [43].

*S. macroglossus* (acetone and water), *T. riparia* (dichloromethane), and *N. oleander* (water) exhibited the lowest MICs (0.16 mg/mL), comparable to rifampicin. The rifampicin MIC obtained here aligns with reported values for *M. smegmatis*, acknowledging strain and methodological variability [44]. Notably, the results for *T. elegans* differ from those of Luo et al. [45], who reported higher potency against various Mycobacterium species; this variation may be attributed to geographic or seasonal influences on phytochemical expression. All extracts were bacteriostatic (MBC > 2.5 mg/mL), suggesting growth-suppressive mechanisms rather than direct bactericidal action.

Interaction studies revealed that while most combinations were indifferent, pairings such as *T. elegans* with *A. praecox* and *T. camphoratus* with Rifampicin showed notable additive effects. These interactions suggest therapeutic potential where phytochemicals may complement conventional antibiotics [46]. Such additivity likely arises from the ability of plant extracts to destabilize the mycobacterial cell wall, thereby facilitating the intracellular entry of rifampicin to its target, DNA-dependent RNA polymerase [46].

Several extracts, including *S. macroglossus* (Sm), *T. riparia* (Tr), and *O. africana* (Oa), demonstrated clear antimycobacterial activity when tested individually, and their combinations predominantly resulted in indifferent or antagonistic interactions. The lack of widespread synergy likely reflects overlapping targets, competitive interactions, or phytochemical antagonism within complex mixtures [47,48]. Nonetheless, the additive interactions provide a foundation for further exploration of combination-based therapeutic strategies. Consistent with this interpretation, the improved activity observed with *T. elegans* combinations is supported by Pallant et al. [49], who reported additive effects between the alkaloidal fraction of *T. elegans* and antibiotics, including ampicillin, ciprofloxacin, and isoniazid, resulting in reduced inhibitory concentrations.

Growth kinetic analyses corroborated the bacteriostatic nature of the extracts, revealing delayed exponential growth rather than complete inhibition. Extracts with lower MICs caused the greatest growth delay, whereas weaker extracts produced partial suppression. Previous studies have reported interactions between volatile and non-volatile fractions of *T. camphoratus* influencing antimicrobial activity [50]. Notably, *L. ocymifolia* and *O. africana* caused the greatest reduction in growth rate. Extracts rich in phenolics and terpenoids are known to disrupt membrane integrity, alter permeability, and impair metabolic processes, leading to slowed replication without immediate lethality [51,52,53]. This bacteriostatic profile is particularly relevant for studying persistence-associated phenotypes, including biofilm formation.

The mechanisms underlying these activities were investigated via cell constituent release and respiratory chain dehydrogenase assays. Increased leakage of nucleic acids (>60%) and proteins (<40%) from *M. smegmatis* (Figure 3A,B) indicates compromised cell envelope integrity. This selective permeability disruption, rather than total lysis, is a hallmark of membrane-targeting antimicrobials [54,55]. Rifampicin has well-established potent antimycobacterial activity against *M. smegmatis* and acts through the inhibition of DNA-dependent RNA polymerase [56], while isoniazid, a reference antitubercular drug, targets mycolic acid synthesis following activation by KatG, highlighting the importance of the mycobacterial cell envelope as a vulnerable therapeutic target [54,57]. Although the present study does not imply similar target specificity, the observed membrane perturbation aligns with the concept that compromising cell envelope integrity disrupts essential physiological processes in mycobacteria. Similar findings have been reported by Mugayi and Mukanganyama [58], reinforcing membrane-associated mechanisms.

In addition to structural damage, extracts significantly reduced respiratory chain dehydrogenase activity (Figure 3C). Notably, *L. ocymifolia* exhibited pronounced inhibition even at sub-MIC concentrations, suggesting metabolic suppression. Dehydrogenases, such as type II NADH dehydrogenase (NDH-2), are critical for maintaining the proton motive force required for ATP synthesis [59]. Inhibition of these enzymes leads to energy depletion and growth arrest. Flavonoids and terpenoids are known to destabilize lipid bilayers and collapse the proton gradient, synergistically promoting metabolic dysfunction and leakage [60,61]. Collectively, these findings indicate that the extracts act through multi-target mechanisms involving both envelope disruption and metabolic inhibition of *M. smegmatis*.

Importantly, anti-virulence activity in this study is defined as interference with biofilm-associated phenotypes rather than inhibition of host-specific virulence factors [62,63]. Biofilm formation contributes to mycobacterial persistence and antimicrobial tolerance. Extracts achieving ≥ 50% were considered to exhibit good antibiofilm activity, and percentage inhibition rates < 0 were considered to have no antibiofilm activity but enhance biofilm development [64]. The extracts demonstrated activity across multiple biofilm developmental stages, including inhibition of initial attachment, prevention of biofilm formation, and partial eradication of mature biofilms. Initial attachment was strongly inhibited (62–98%), with *N. oleander* and *O. africana* showing near-complete prevention (93–100%), consistent with previous reports [65]. Interestingly, *S. macroglossus* promoted attachment at certain concentrations but inhibited later biofilm development, suggesting complex surface interactions.

Established biofilms were more resistant; however, *N. oleander*, *O. africana*, and *S. macroglossus* showed improved eradication at sub-MIC concentrations, indicating disruption of EPS integrity or biofilm signaling pathways rather than direct killing [66]. Energy depletion and membrane disruption likely impair biofilm matrix production and adhesin expression, effects previously linked to polyphenolics [67]. By limiting the transition from a planktonic to a sessile state, these extracts demonstrate clear anti-virulence potential, effectively disarming the bacteria’s ability to establish protective communities. Antimotility assays further supported this mechanism. Although mycobacteria lack flagella, sliding motility mediated by surface lipids is closely linked to biofilm initiation. Several extracts significantly reduced sliding motility, indicating disruption of lipid-dependent surface translocation [68]. Together, these findings demonstrate that the extracts effectively modulate persistence-related behaviors, highlighting their anti-virulence potential rather than classical bactericidal activity.

## 5. Conclusions

This study demonstrates that selected medicinal plant extracts exert significant effects against *M. smegmatis*, influencing both growth and key virulence-associated phenotypes. Extracts of *S. macroglossus*, *N. oleander*, and *T. riparia* showed the strongest growth inhibition, with MIC values comparable to rifampicin, and displayed bacteriostatic activity as confirmed by growth kinetics. Mechanistic assays revealed that these extracts disrupt membrane integrity, induce leakage of intracellular constituents, and inhibit respiratory chain dehydrogenase activity, indicating interference with essential physiological processes required for bacterial energy generation and survival.

While only one batch per medicinal plant was analyzed, this approach aligns with the exploratory nature of early-stage antimicrobial screening, where the primary aim is to identify bioactive leads rather than establish product standardization. Future work should focus on cytotoxicity profiling, bioassay-guided isolation, and compound-level analysis, as well as testing against *M. tuberculosis* H37Rv and clinical isolates, to elucidate precise structure–activity relationships and evaluate the potential of these extracts as lead candidates for antimycobacterial or adjunctive anti-virulence therapies.

## Figures and Tables

**Table 1:** Total phenolic, tannin, flavonoid, and flavonol contents of the plant extracts.

Plant Extracts (Acetone)	Total Phenolic Content (mg GAE/g Extract)	Total Tannin Content (mg GAE/g Extract)	Total Flavonoid Content (mg QE/g Extract)	Total Flavonols Content (mg QE/g Extract)
Ap	6.09 ± 0.26 ^b^	0.20 ± 0.004 ^b^	1.72 ± 0.04 ^b^	5.62 ± 0.71 ^b^
Ch	24.59 ± 0.08 ^f^	1.91 ± 0.02 ^f^	10.09 ± 0.19 ^g^	14.39 ± 0.67 ^d^
Lo	12.05 ± 0.79 ^d^	0.41 ± 0.004 ^d^	7.23 ± 0.39 ^f^	23.71 ± 0.53 ^e^
No	6.04 ± 0.23 ^b^	0.30 ± 0.005 ^c^	2.57 ± 0.07 ^c^	1.31 ± 0.89 ^a^
Pm	11.09 ± 0.16 ^c^	0.34 ± 0.013 ^c^	5.18 ± 0.06 ^e^	22.97 ± 0.97 ^e^
Oa	11.41 ± 0.03 ^c,d^	0.49 ± 0.27 ^e^	3.43 ± 0.06 ^d^	26.99 ± 0.90 ^f^
Sm	20.24 ± 0.07 ^e^	1.07 ± 0.008 ^f^	12.56 ± 0.10 ^h^	27.65 ± 0.80 ^f^
Te	3.09 ± 0.14 ^a^	0.11 ± 0.005 ^a^	1.23 ± 0.06 ^a^	43.19 ± 0.24 ^g^
Tc	28.94 ± 0.53 ^g^	2.19 ± 0.009 ^g^	30.61 ± 0.09 ^i^	10.34 ± 0.48 ^c^
Tr	5.90 ± 0.12 ^b^	0.21 ± 0.003 ^b^	2.78 ± 0.09 ^c^	1.18 ± 0.44 ^a^

Key: GAE—gallic acid equivalence, QE—quercetin equivalence, Ap—*Agapanthus praecox*, Ch—*Combretum hereroense*, Lo—*Leonotis ocymifolia*, No—*Nerium oleander*, Pm—*Polygala myrtifolia*, Oa—*Olea europaea* supsp. *africana*, Sm—*Senecio macroglossus*, Te—*Tabernaemontana elegans*, Tc—*Tarchonanthus camphoratus*, and Tr—*Tetradenia raparia*. Different letters represent statistically significant differences (*p* < 0.05), and same letters indicate insignificant differences (*p* > 0.05).

**Table 2:** Antioxidant activity of the plant extracts.

Samples	DPPH Scavenging Activity	r^2^	Ferric Reducing Power	r^2^
Ap	789.83	0.99	1296.67	0.99
Ch	50.21	0.99	63.15	0.99
Lo	652.24	0.96	757	0.99
No	490.79	0.99	848.2	0.97
Pm	464.25	0.99	793.8	0.98
Oa	332.35	0.96	773.4	0.98
Sm	247.75	0.98	367.3	0.98
Te	1456	0.97	2058	0.98
Tc	38.73	0.99	195.6	0.99
Tr	466.90	0.99	654.33	0.99
Ascorbic acid	33.31	0.97	48.70	0.99

Key: Ap—*Agapanthus praecox*, Ch—*Combretum hereroense*, Lo—*Leonotis ocymifolia*, No—*Nerium oleander*, Pm—*Polygala myrtifolia*, Oa—*Olea europaea* supsp. *africana*, Sm—*Senecio macroglossus*, Te—*Tabernaemontana elegans*, Tc—*Tarchonanthus camphoratus*, Tr—*Tetradenia raparia*.

**Table 3:** Antimycobacterial activities of selected plant extracts against *M. smegmatis*.

Plants	H			D			A			M			W		
	MIC	MBC	TA	MIC	MBC	TA	MIC	MBC	TA	MIC	MBC	TA	MIC	MBC	TA
Ap	2.5	>2.5	16.80	1.04	>2.5	52.88	1.25	>2.5	29.60	1.25	>2.5	92.80	2.5	>2.5	32.00
Ch	0.63	>2.5	123.81	0.63	>2.5	130.16	0.63	>2.5	138.10	1.25	>2.5	160.80	0.63	>2.5	280.95
Lo	1.25	>2.5	36.80	0.63	>2.5	158.73	0.52	>2.5	130.77	1.25	>2.5	85.60	1.25	>2.5	65.60
No	2.5	>2.5	11.20	1.25	>2.5	60.00	1.25	>2.5	47.20	1.25	>2.5	79.20	0.16	>2.5	518.75
Pm	2.5	>2.5	20.00	0.63	>2.5	185.71	0.63	>2.5	109.52	1.25	>2.5	160.80	1.25	>2.5	133.60
Oa	2.5	>2.5	4.00	2.5	>2.5	20.40	0.63	>2.5	55.56	0.31	>2.5	367.74	0.63	>2.5	188.89
Sm	2.5	>2.5	74.00	0.63	>2.5	215.87	0.16	>2.5	112.50	0.63	>2.5	247.60	0.16	>2.5	806.25
Te	2.5	>2.5	21.20	0.83	>2.5	155.42	2.5	>2.5	46.40	1.25	>2.5	94.40	>2.5	>2.5	-
Tc	2.5	>2.5	15.20	1.25	>2.5	63.20	0.63	>2.5	109.52	1.25	>2.5	66.40	0.63	>2.5	150.79
Tr	2.5	>2.5	30.00	0.16	>2.5	187.50	0.83	>2.5	112.05	2.5	>2.5	145.60	2.5	>2.5	24.00
Rif	0.16														

Key: MIC—minimum inhibitory concentration (mg/mL), MBC—minimum bactericidal concentration (mg/mL), TA—total activity (mL/g), H—hexane, D—dichloromethane, A—acetone, M—methanol, W—water, Ap—*Agapanthus praecox*, Ch—*Combretum hereroense*, Lo—*Leonotis ocymifolia*, No—*Nerium oleander*, Pm—*Polygala myrtifolia*, Oa—*Olea europaea* supsp. *africana*, Sm—*Senecio macroglossus*, Te—*Tabernaemontana elegans*, Tc—*Tarchonanthus camphoratus,* Tr—*Tetradenia raparia*, Rif—rifampicin.

**Table 4:** Combinational antimycobacterial effects of selected plant extracts in combination with each other and with rifampicin against *M. smegmatis*.

Combination	FIC (A)	FIC (B)	FIC Index (ΣFIC)	Outcome
Ap + Ch	0.50	1.00	1.50	Indifferent
Ap + Lo	0.50	1.21	1.72	Indifferent
Ap + No	1.66	1.66	3.33	Indifferent
Ap + Pm	0.50	1.00	1.50	Indifferent
Ap + Oa	0.50	1.00	1.50	Indifferent
Ap + Sm	0.34	2.63	2.96	Indifferent
Ap + Te	0.50	0.25	0.76	Additive
Ap + Tc	0.50	1.00	1.50	Indifferent
Ap + Tr	0.50	0.76	1.26	Indifferent
Ch + Lo	1.00	1.21	2.21	Indifferent
Ch + No	1.98	1.00	2.98	Indifferent
Ch + Pm	1.00	1.00	2.00	Indifferent
Ch + Oa	1.00	1.00	2.00	Indifferent
Ch + Sm	0.49	1.94	2.43	Indifferent
Ch + Te	1.00	0.25	1.25	Indifferent
Ch + Tc	1.00	1.00	2.00	Indifferent
Ch + Tr	1.00	0.76	1.76	Indifferent
Lo + No	1.21	0.50	1.72	Indifferent
Lo + Pm	2.40	1.98	4.39	Antagonistic
Lo + Oa	1.21	1.00	2.21	Indifferent
Lo + Sm	0.81	2.63	3.43	Indifferent
Lo + Te	2.40	0.50	2.90	Indifferent
Lo + Tc	1.21	1.00	2.21	Indifferent
Lo + Tr	1.19	0.75	1.94	Indifferent
No + Pm	1.00	1.98	2.98	Indifferent
No + Oa	1.00	1.98	2.98	Indifferent
No + Sm	0.25	1.94	2.19	Indifferent
No + Te	0.50	0.25	0.76	Additive
No + Tc	0.50	1.00	1.50	Indifferent
No + Tr	0.50	0.76	1.26	Indifferent
Pm + Oa	1.00	1.00	2.00	Indifferent
Pm + Sm	0.49	1.94	2.43	Indifferent
Pm + Te	1.32	0.33	1.65	Indifferent
Pm + Tc	1.98	1.98	3.97	Indifferent
Pm + Tr	1.98	1.51	3.49	Indifferent
Oa + Sm	0.49	1.94	2.43	Indifferent
Oa + Te	1.00	0.25	1.25	Indifferent
Oa + Tc	1.00	1.00	2.00	Indifferent
Oa + Tr	1.00	0.76	1.76	Indifferent
Sm + Te	3.94	0.25	4.19	Antagonistic
Sm + Tc	3.94	1.00	4.94	Antagonistic
Sm + Tr	3.94	0.76	4.70	Antagonistic
Te + Tc	0.25	1.00	1.25	Indifferent
Te + Tr	1.00	3.01	4.01	Antagonistic
Tc + Tr	1.00	0.76	1.76	Indifferent
Combinational effects with the positive control				
Ap + R	0.50	3.94	4.44	Antagonistic
Ch + R	0.25	1.00	1.25	Indifferent
Lo + R	1.21	3.94	5.15	Antagonistic
No + R	0.13	1.00	1.13	Indifferent
Pm + R	1.00	3.94	4.94	Antagonistic
Oa + R	1.00	3.94	4.94	Antagonistic
Sm + R	1.94	1.94	3.88	Indifferent
Te + R	0.33	5.19	5.52	Antagonistic
Tc + R	0.19	0.75	0.94	Additive
Tr + R	1.51	7.81	9.32	Antagonistic

Key: FICA—fractional inhibitory concentration of extract A, FICB—fractional inhibitory concentration of extract B, ΣFIC—the sum of FICA and FICB, Ap—*Agapanthus praecox*, Ch—*Combretum hereroense*, Lo—*Leonotis ocymifolia*, No—*Nerium oleander*, Pm—*Polygala myrtifolia*, Oa—*Olea europaea* supsp. *africana*, Sm—*Senecio macroglossus*, Te—*Tabernaemontana elegans*, Tc—*Tarchonanthus camphoratus*, Tr—*Tetradenia raparia*, R—rifampicin.

**Table 5:** The antimotility activity of selected plant extracts expressed as percentage inhibition.

Plants	MIC	½ MIC
Rifampicin	100 ± 0.00	100 ± 0.00
*Leonotis ocymifolia*	56.43 ± 1.01	41 ± 2.63
*Nerium oleander*	100 ± 0.00	70.71 ± 1.01
*Olea europaea* subsp *africana*	70 ± 2.02	12.57 ± 2.42
*Senecio macroglossus*	41.79 ± 1.52	56 ± 1.62
*Tetradenia raparia*	27.29 ± 1.82	99.29 ± 1.01

Key: MIC—minimum inhibitory concentration. Values expressed as mean of triplicate ± standard deviation.

## Data Availability

The original contributions presented in this study are included in the article. Further inquiries can be directed to the corresponding author.

## Abbreviations

The following abbreviations are used in this manuscript:

ANOVA	Analysis of variance
EC50	Half maximal effective concentration
mg GAE/g	Milligrams of gallic acid equivalents per gram of extract
mg QE/g	Milligrams of quercetin equivalents per gram of extract
OD600	Optical density at 600 nanometers
